# Coexistence of *TERT* Promoter Mutations and the *BRAF* V600E Alteration and Its Impact on Histopathological Features of Papillary Thyroid Carcinoma in a Selected Series of Polish Patients

**DOI:** 10.3390/ijms19092647

**Published:** 2018-09-06

**Authors:** Dagmara Rusinek, Aleksandra Pfeifer, Jolanta Krajewska, Malgorzata Oczko-Wojciechowska, Daria Handkiewicz-Junak, Agnieszka Pawlaczek, Jadwiga Zebracka-Gala, Malgorzata Kowalska, Renata Cyplinska, Ewa Zembala-Nozynska, Mykola Chekan, Ewa Chmielik, Aleksandra Kropinska, Roman Lamch, Beata Jurecka-Lubieniecka, Barbara Jarzab, Agnieszka Czarniecka

**Affiliations:** 1 Department of Nuclear Medicine and Endocrine Oncology, Maria Sklodowska-Curie Institute, Oncology Center, Gliwice Branch, Wybrzeze Armii Krajowej 15, 44-101 Gliwice, Poland; Aleksandra.Pfeifer@io.gliwice.pl (A.P.); Jolanta.Krajewska@io.gliwice.pl (J.K.); Malgorzata.Oczko-Wojciechowska@io.gliwice.pl (M.O.-W.); Daria.Handkiewicz-Junak@io.gliwice.pl (D.H.-J.); Agnieszka.Pawlaczek@io.gliwice.pl (A.P.); Jadwiga.Zebracka-Gala@io.gliwice.pl (J.Z.-G.); Malgorzata.Kowalska@io.gliwice.pl (M.K.); Renata.Cyplinska@io.gliwice.pl (R.C.); Aleksandra.Kropinska@io.gliwice.pl (A.K.); Beata.Jurecka-Lubieniecka@io.gliwice.pl (B.J.-L.); Barbara.Jarzab@io.gliwice.pl (B.J.); 2 Tumor Pathology Department, Maria Sklodowska-Curie Institute, Oncology Center, Gliwice Branch, Wybrzeze Armii Krajowej 15, 44-101 Gliwice, Poland; Ewa.Zembala-Nozynska@io.gliwice.pl (E.Z.-N.); Mykola.Chekan@io.gliwice.pl (M.C.); Ewa.Chmielik@io.gliwice.pl (E.C.); Roman.Lamch@io.gliwice.pl (R.L.); 3 Department of Oncological and Reconstructive Surgery, Maria Sklodowska-Curie Institute, Oncology Center, Gliwice Branch, Wybrzeze Armii Krajowej 15, 44-101 Gliwice, Poland; Agnieszka.Czarniecka@io.gliwice.pl

**Keywords:** *TERT*p mutation, *BRAF* V600E, papillary thyroid cancer

## Abstract

*TERT* promoter (*TERT*p) mutations are important factors in papillary thyroid carcinomas (PTCs). They are associated with tumor aggressiveness, recurrence, and disease-specific mortality and their use in risk stratification of PTC patients has been proposed. In this study we investigated the prevalence of *TERT*p mutations in a cohort of Polish patients with PTCs and the association of these mutations with histopathological factors, particularly in coexistence with the *BRAF* V600E mutation. A total of 189 consecutive PTC specimens with known *BRAF* mutational status were evaluated. *TERT*p mutations were detected in 8.5% of cases (16/189) with the C228T mutation being the most frequent. In six of the PTC specimens (3.2%), four additional *TERT*p alterations were found, which included one known polymorphism (rs2735943) and three previously unreported alterations. The association analysis revealed that the *TERT*p hotspot mutations were highly correlated with the presence of the *BRAF* V600E mutation and their coexistence was significantly associated with gender, advanced patient age, advanced disease stage, presence of lymph node metastases, larger tumor size, and tumor-capsule infiltration. While correlations were identified, the possibility of *TERT*p mutations being key molecular modulators responsible for PTC aggressiveness requires further studies.

## 1. Introduction

Despite excellent overall survival in most cases of papillary thyroid carcinoma (PTC), recurrent disease is diagnosed during the 10-year period following initial treatment in 15%, 22%, 46.4%, and 66.7% of patients with TNM stage I, II, III, and IV, respectively. PTC may be a cause of death, even in patients with clinically indolent tumors [1]. Distant metastases of PTC are a strong predictor of a poor outcome, including disease-specific mortality [2,3]. However, neck lymph node metastases (LNM) are also considered to be a predictor of persistent or recurrent disease [4]. Molecular markers of poor prognosis also exist and include genetic alterations associated with PTC. The *BRAF* V600E mutation occurs in about 50% of PTC cases and has been identified in a number of studies to be related to an advanced disease stage, extrathyroidal invasion, or the presence of LNM [5]. It is also reported that the presence of *BRAF* mutation is associated with a decreased expression of thyroid specific genes like *NIS* and *TPO*, what results in the loss of sensitivity to radioiodine [6]. However, there are also contradicting studies, in which no significant association between *BRAF* V600E and poor prognosis is demonstrated [7,8].

Recent reports provide new data on the molecular background of PTC, especially as it relates to its aggressive phenotype. Mutations in the telomerase reverse transcriptase promoter (*TERT*p) have been detected in a number of solid cancers, including thyroid cancers [9,10,11]. *TERT* encodes the catalytic subunit of telomerase, the enzyme responsible for extending telomeres and thereby preventing replicative senescence. TERT, together with its RNA partner telomerase TERT component (TERC), demonstrates telomerase reverse transcriptase activity and adds tandem repeats of TTAGGG sequence to the end of chromosomes [12]. As demonstrated in many studies, telomerase is highly expressed in germ cells and stem cells, but is repressed and not transcribed in most somatic cells [13]. However, the reactivation of telomerase has been observed in numerous human cancers, which prevents telomere shortening and thereby provides cancer cells an unlimited replicative potential [14]. Although telomere elongation is the primary function of reactivated telomerase, it has a number of other effects, among them: growing cancer cell proliferation, resistance to apoptosis and antigrowth signals, increased angiogenesis and metastatic potential [15]. Considering all the above-mentioned processes, in which *TERT* is involved, it has been proposed to treat *TERT* as an oncogene that is able to act in a telomere-independent manner [15].

In numerous malignancies, including follicular cell derived thyroid cancers (TCs), two hotspot mutations within *TERT*p have been described, chr5, 1,295,228 C->T (C228T; hg19), and chr5, 1,295,250 C->T (C250T; hg19), which correspond to positions −124 and −146 bp, respectively, and are located upstream from the ATG start codon of *TERT* [16]. The prevalence of these two mutations in TCs, of which C228T is more frequent, seems to correlate with the degree of aggressiveness in various TC subtypes. They have been shown to be present in 11.3%, 17.1%, 14.6%, 43.2%, and 40.1% of PTC, follicular thyroid carcinoma (FTC), Hürthle cell carcinoma (HCC), poorly differentiated thyroid carcinoma (PDTC), and anaplastic thyroid carcinoma (ATC), respectively [12]. A similar trend in the frequency of *TERT*p mutations is observed in particular histopathological PTC variants with the highest prevalence being seen in tall-cell PTC variant known for its aggressive phenotype [9]. The presence of these genetic alterations has also been detected in papillary thyroid microcarcinomas (PTMC), but their prevalence does not exceed 4.7% and no relationship to unfavorable clinical features is observed [17]. Interestingly, *TERT*p mutations were widely reported as associated with the presence of the *BRAF* V600E mutation in human cancers, mainly in TC and melanoma [11,12,18,19]. Alone, these mutations display a modest effect; however the coexistence of *BRAF* V600E and *TERT*p mutations is associated with a higher cancer aggressiveness, which is reflected by lymph node metastases, distant metastases, advanced tumor stage, recurrence, and even disease-specific mortality of PTC patients [20,21]. According to a recent meta-analysis concerning PTCs, the prevalence of the coexistence of the *BRAF* V600E and *TERT*p mutations is 7.7% (145/1892) [12]. This frequency corresponds to the percentage of PTCs with poorest clinical outcome, estimated as less than 10%. These data indicate the *BRAF* V600E and *TERT*p mutations as an oncogene tandem underlying progression and aggressiveness of thyroid cancers. Similar clinical consequences are observed regarding the coexistence of *RAS* mutations and *TERT*p mutations [12,21]. This makes *TERT* a potential prognostic marker that may be beneficial for risk stratification of patients with PTC. However, several studies investigating the prevalence of *TERT*p mutations in TCs have shown significant differences in the frequency of *TERT*p alterations in different countries with rates of 7.5–25.5% and 13.8–36.4% for PTC and FTC, respectively [22].

In the current study we analyzed the prevalence of *TERT*p mutations as well as their potential associations with the *BRAF* V600E alteration and histopathological features in selected series of Polish PTC patients. Moreover, the differences between the presence of a single mutation and the coexistence of *TERT*p and *BRAF* 600 codon alterations and their relation to particular histopathological PTC features were analyzed. 

## 2. Results

### 2.1. TERT Promoter Status in PTCs and Its Relation to the Presence of the BRAF V600E Mutation

**Figure 1:**
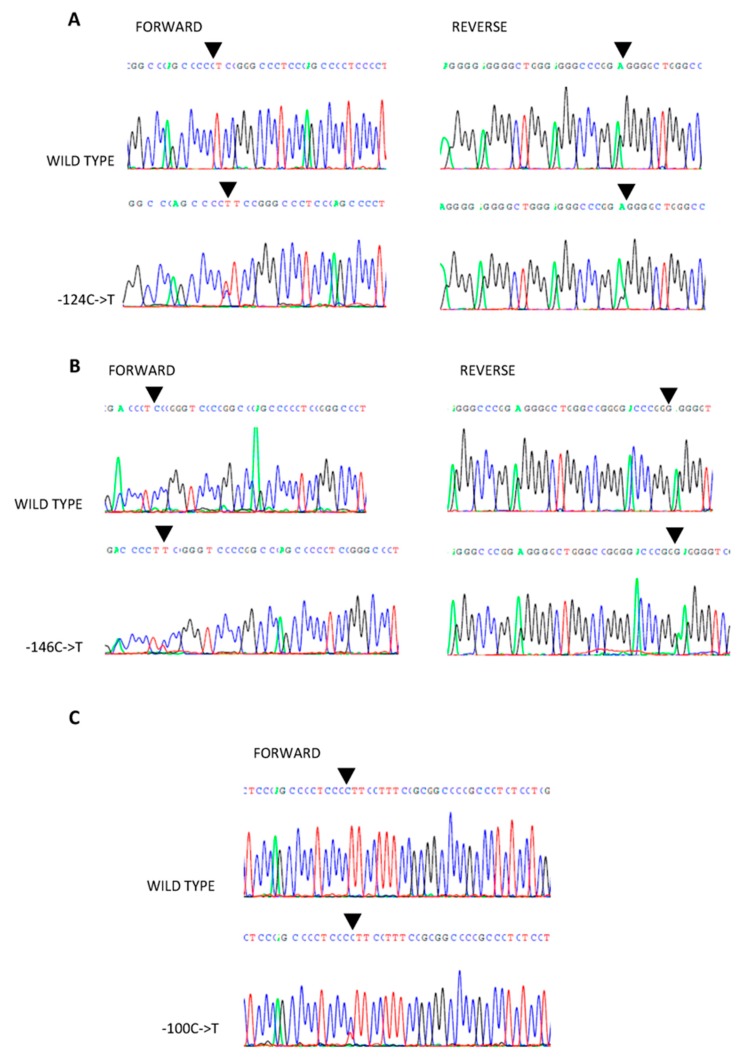
Known *TERT* promoter *(TERT*p) alterations detected in the analyzed papillary thyroid carcinoma (PTC) cohort. (**A**) Mutation −124 C->T (C228T); (**B**) mutation −146 C->T (C250T); (**C**) alteration −100 C->T (rs2735943). The black triangles point to the nucleotide positions of the nucleotide changes.

**Figure 2:**
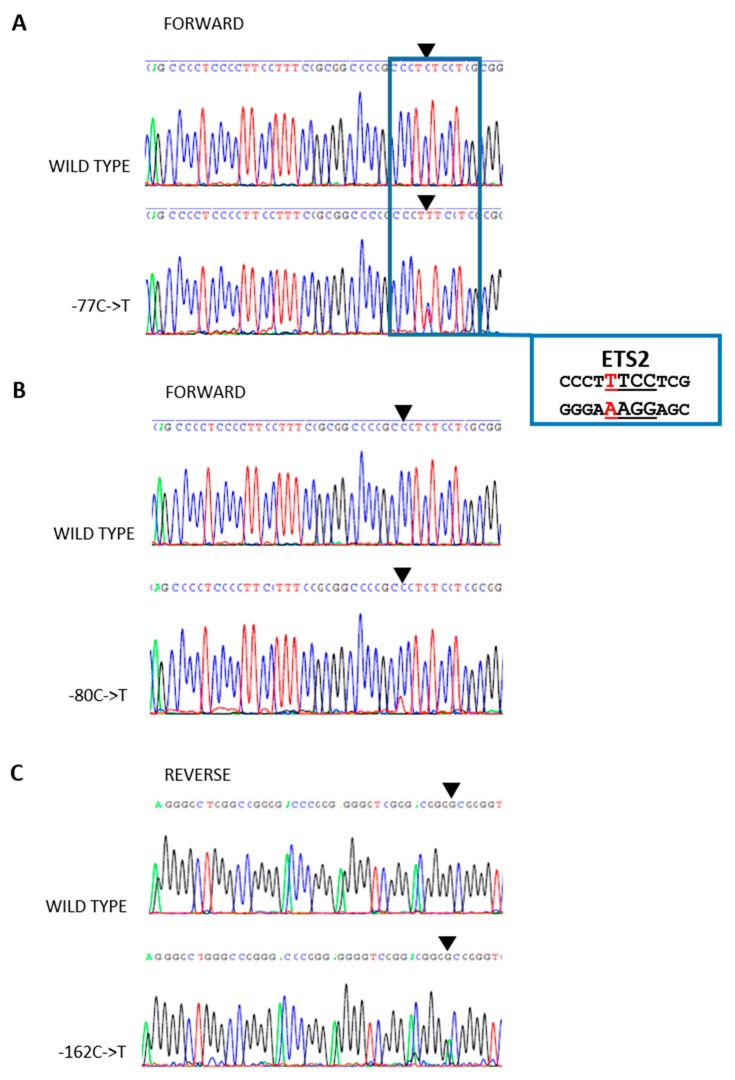
New *TERT* promoter (*TERT*p) alterations detected in the analyzed papillary thyroid carcinoma (PTC) cohort. (**A**) Alteration −77 C->T, which created a putative consensus binding site (GGAA) for transcription factor ETS2; (**B**) alteration −80 C->T; (**C**) alteration −162 C->T. The black triangles point to the nucleotide positions of the nucleotide substitutions.

We evaluated *TERT*p in 189 consecutive PTC tumor specimens for the presence of two hotspot mutations, C228T and C250T. In total, 16 of these 189 PTC specimens (8.5%) were positive for the C228T or C250T *TERT*p mutations. Of the specimens analyzed, 13 harbored the C228T mutation (81%; Figure 1A) and the remaining three PTC specimens had the C250T mutation (Figure 1B). In the tumor from one patient, who was positive for the *BRAF* V600E mutation, a known polymorphism within *TERT*p was found (rs2735943; −100 C->T; Figure 1C). Three new *TERT*p mutations were also identified in five of the PTC specimens. None of the *TERT*p mutations detected coexisted with each other in any of the samples analyzed. The new mutations found in *TERT*p were located at positions −162 bp, −80 bp, and −77 bp, upstream of the ATG start codon of *TERT*, and all involved a substitution of cytosine to thymine (Figure 2A–C). None of the new nucleotide substitutions, detected in the specimens from our PTC cohort, have been previously reported. Because of the lack of knowledge regarding whether we were dealing with a mutation or a polymorphism, and since one PTC specimen had the rs2735943 polymorphism, which has an unknown frequency, the new *TERT*p mutations were analyzed further as a separate set of samples and excluded from the *TERT*p-positive cohort, which consisted only of PTC specimens harboring the hotspot mutations C228T or C250T.

In order to analyze the relationship of *TERT*p hotspot mutations with the presence of the *BRAF* V600E mutation, the cases with *TERT*p alteration other than C228T or C250T were excluded from the PTC dataset. This resulted in 87 *BRAF*-positive and 96 *BRAF*-negative samples being analyzed. A significant association between *TERT*p hotspot mutations and the presence of *BRAF* mutation was identified. Thirteen of the 16 *TERT*p-positive PTC specimens that contained the C228T mutation and all that contained the C250T mutation also harbored the *BRAF* V600E mutation (81.2%; *p* = 0.007; Table 1). The remaining three *TERT*p-positive PTC specimens were *BRAF* V600E-negative and all contained the C228T mutation. In contrast, the *BRAF* V600E mutation was found in 74 *TERT*p-negative cases (74/167; 44.3%; Table 1). 

### 2.2. Association of the BRAF V600E Mutation and TERTp Alterations with Histopathological Factors in the Cohort of Polish Patients with PTC

In order to analyze the cohort of Polish patients with PTC for a possible relationship between each of the investigated molecular events, the samples were divided into four subgroups, (1) no detected mutation within the *BRAF* 600 codon or *TERT*p; (2) positive for the *BRAF* V600E mutation but no *TERT*p alterations; (3) positive for the *BRAF* V600E mutation and one of the hotspot *TERT*p mutations; and (4) a *TERT*p alterations present other than C228T or C250T. Subgroups 2–4 were compared to subgroup 1 to determine how the presence of the analyzed alterations was related to particular histopathological PTC features, both individually or together (Table 2).

The *BRAF* V600E mutation alone was significantly associated with more advanced age of the patients (*p* = 0.024), larger tumor size (*p* < 0.0001), more advanced disease stage (*p* = 0.002), and infiltration of the tumor capsule (*p* = 0.0007). The *TERT*p hotspot mutations alone were significantly associated only with advanced age of patients (*p* = 0.005) and histopathological PTC variant (*p* = 0.041); however, the number of cases in the cohort was too small to interpret these results with a high degree of confidence. Coexistence of the *BRAF* V600E mutation and *TERT*p hotspot mutations, demonstrated an association with gender (*p* = 0.006), advanced age of the patients (*p* < 0.0001), advanced disease stage (*p* < 0.0001), the presence of lymph nodes metastases (*p* < 0.0001), larger tumor size (*p* < 0.0001), and infiltration of the tumor capsule (*p* = 0.0007). The results are summarized in Table 2.

With respect to the subgroup of PTC specimens with *TERT*p alterations other than the hotspot mutations, no association with any of the analyzed factors was identified. However, the small number of samples in that group (n = 6) does not justify drawing any unequivocal conclusions. Also, it was not possible to determine the association of the studied alterations with distant metastases since none of PTC cases in the cohort demonstrated distant metastases at the time of diagnosis. 

### 2.3. Further Analysis of the Presence of TERTp Alterations in a Subgroup of Patients with PTMC

Among 80 PTMC specimens, only one tumor harbored the *TERT*p hotspot mutation C228T, while two other specimens displayed unknown *TERT*p alterations −77 C->T and −162 C->T, with the latter one also being positive for the *BRAF* V600E mutation. In contrast to PTMC specimens containing a single alteration, which was consistent with classical PTC, the single case that harbored the *BRAF* mutation and the −162 C->T *TERT*p alteration was diagnosed as a follicular PTC variant. None of the three *TERT*p-positive PTMC specimens demonstrated angioinvasion, infiltration of tumor capsule, multifocality, or LNM. Twenty of 80 PTMC specimens contained the *BRAF* V600E mutation. However, infiltration of the tumor capsule was present in only 20% (4/20) of the *BRAF*-positive cases. These specimens were taken from the oldest patients in the PTMC subgroup. No other histopathological factors related to poor prognosis were observed.

## 3. Discussion

Since their discovery, somatic *TERT*p mutations have been extensively studied in a number of cancers, including melanomas, glioblastomas, clear cell renal carcinomas and nonclear cell renal carcinomas, non-small cell lung cancer, and TCs [10,12,23,24,25]. It has been demonstrated that the frequency of *TERT*p mutations is higher in more aggressive types of TC, as well as in histopathological PTC variants, and the presence of the mutations correlate with a poorer outcome [9,12]. Moreover, several investigators suggest that the presence of these alterations in PTCs is associated with particularly aggressive clinicopathological factors, such as greater tumor size, extrathyroidal invasion, stage III or IV disease, lymph node metastases, and distant metastases [26,27].

Taking into consideration the divergence observed in the frequency of *TERT*p mutations in PTC between different countries and scarce data concerning the impact of *TERT*p mutations alone on PTC histopathological features we decided to investigate Polish PTC patients. Regarding the well-documented association between the presence of *TERT*p and *BRAF* V600E mutations we analyzed *TERT*p in PTCs with known *BRAF* 600 codon status. We also performed a number of comparative analyses to identify a potential role of these two molecular markers alone or their coexistence in PTC carcinogenesis. The *TERT*p hotspot mutations were detected in 16 of the 189 consecutive analyzed PTC specimens (8.5%). However, if we excluded PTMCs and focused only on PTCs, the frequency of *TERT*p mutations increased up to 13.8% (15/109). Such observation is in concordance with previous studies [12]. In our study, one patient diagnosed with PTMC harbored the *TERT*p hotspot mutation C228T but not the *BRAF* V600E mutation. Consistent with the findings of de Biase and coauthors [17], *TERT*p mutations were present, but relatively uncommon in PTMC specimens at a frequency of 4.7%, and did not correlate with unfavorable clinical features. Similarly, our only case of PTMC with the C228T *TERT*p mutation did not demonstrate extrathyroidal invasion, angioinvasion, or lymph node metastases. In addition to the known hotspot mutations in *TERT*p, we also detected another known alteration, previously reported in dbSNP (rs2735943). Because its frequency is not known it was treated in our study like the three new *TERT*p mutations we identified, since there was no certainty whether it was a polymorphism or mutation. The analyses revealed that these four *TERT*p alterations (−100 C->T; −162 C->T; −80 C->T; −77 C->T) were not associated with any of analyzed clinical factors. However, we cannot exclude their impact on PTC carcinogenesis and further studies are needed to determine their role. It is noteworthy that one of the novel nucleotide substitutions (−77 C->T) created a putative consensus binding site (GGAA) for ETS (E26 transformation-specific) transcription factors; the same as that created by C228T and C250T hotspot mutations. It is believed that creating such additional binding sites for transcription factors is a mechanism of action for C228T and C250T, related to increased *TERT* expression [28]. 

In the cohort of Polish patients with PTC, analyzed in the current study, the *TERT*p hotspot mutations were more prevalent in the *BRAF* V600E-positive specimens compared to *BRAF* V600E-negative specimens (81.2%; *p* = 0.007) and the *BRAF* V600E mutation was less frequent in *TERT*p-negative PTC specimens. Our findings are consistent with a previous report [12]. However, there are some contradicting data showing no correlation in the occurrence of these two molecular events [29]. Contrary results were obtained by Landa and coworkers [30], who observed that *TERT*p mutations were less common in well-differentiated PTC specimens harboring *BRAF* or *RAS* mutations. However, in PDTC specimens or ATC specimens, *BRAF*-positive or *RAS*-positive cases are enriched by *TERT*p alterations. This observation supports the belief that *TERT*p mutations may be useful as potential prognostic markers for TC.

We analyzed whether *TERT*p hotspot mutations were related to any poor prognostic factors in Polish patients with PTC. We determined that these mutations alone were associated only with advanced age at the time of PTC diagnosis and with a histopathological PTC variant (oxyphilic). However, the number of *BRAF*(−)*TERT*p(+) samples was small so these data are limited for their interpretative value. On the other hand, similar results were obtained by Gandolfi et al. [31] who described a significant association between *TERT*p mutations and advanced age, but only in PTC cases with distant metastases. In a study by Shen et al. [21], *TERT*p mutations analyzed individually failed to show any significant association with clinicopathological outcomes. In contrast, the coexistence of the *BRAF* V600E mutation and *TERT*p hotspot mutations is reported to be strongly associated with a number of histopathological factors related to more aggressive clinical course of the disease [21,31]. The same relations were detected in our study with a significant association of the coexistence of *BRAF* and *TERT*p mutations with gender, advanced age of patients, T3 and T4 stage of disease, the presence of lymph node metastases, larger tumor size, and infiltration of the tumor capsule. The role of these two genetic alterations in promoting PTC aggressiveness and poor clinical outcome may be explained by the mechanisms observed at the molecular level. Previous studies indicated a possible molecular interaction between *BRAF* V600E and *TERT*p mutations via *BRAF* V600E-dependent constitutive activation of MAPK pathway leading to activation of ETS transcription factors and their binding to binding motifs, created de novo by the hotspot mutations in *TERT*p, what in consequence would enhance *TERT* activity. However, Liu et al. study from 2018 showed the exact role of *BRAF* and *TERT*p mutations in the expression of *TERT* through the *BRAF* V600E-> MAPK pathway->FOS-> GABP-> *TERT* axis [32]. Moreover, the authors presented that *BRAF* V600E/MAPK pathway also promoted *TERT* expression via *MYC* in *TERT*p mutation-independent manner, but in a much less degree.

According to the previous studies, *TERT*p hotspot mutations were found in more aggressive histopathological PTC variants, like tall cell variant [9]. Our PTC cohort was mainly represented by classical PTCs and follicular variant. There was only one case of tall cell PTC and it was positive for the *BRAF* V600E mutation and *TERT*p hotspot alterations. 

The major limitation of the current study was a small number of cases with more aggressive clinical course of the disease, including no PTC cases with distant metastases. However, while we were not able to identify a possible relation between *TERT*p mutations and more aggressive PTC subtypes, our data did show a strong effect of coexistence of *TERT*p hotspot mutations and the *BRAF* V600E mutation on the development of PTCs with histopathological factors related to poorer outcome. In order to determine the independent effects of *TERT*p mutations on PTC, an analysis of a larger cohort is needed. There is a single study in which only *TERT*p mutations, but not the *BRAF* V600E mutation, were associated with poorer histopathological factors [29]. However, it should be noted that the number of PTC samples with *TERT*p mutations alone and PTCs with double mutations (*TERT*p and *BRAF* mutations) was only four in each group. Thus, the data from such limited sample size should be interpreted with caution. 

So far, the detection of *BRAF* V600E and *TERT*p mutations in TCs is not a routine diagnostic procedure. These alterations are mainly analyzed in fine needle aspiration biopsy material in order to support the cytological examination. According to the ATA recommendations [33] molecular tests may be used to refine risk prior to surgery, assuming that surgical management would change based on a positive test result. As mentioned earlier, *TERT*p mutations were shown to be a helpful tool in PTC risk stratification, however, no standardized therapeutic strategies, based on these two molecular markers, were established.

## 4. Material and Methods

### 4.1. DNA Isolation

The analyzed group of specimens were collected from 189 consecutive Polish patients with PTC treated at the Maria Sklodowska-Curie Institute—Oncology Center, Gliwice Branch in Gliwice, Poland. Among them, 80 (42%; 80/189) of patients were diagnosed with papillary microcarcinoma. The selection of the patients was based on the known mutation status in *BRAF* 600 codon. The study cohort included 166 women and 23 men with the median age at the time of PTC diagnosis of 53 years (range 12 to 83 years). The clinical data are summarized in Table 3. All samples were analyzed for the presence of *TERT*p mutations. The use of human tissue was approved by the institutional Bioethics Committee. Informed written consent was obtained for the use of their tissue for analysis from all patients or their caregivers. All the clinical data were anonymized and de-identified prior to the analysis.

### 4.2. DNA Isolation

Total DNA was extracted from 10 μm-thick sections of formalin-fixed and paraffin-embedded (FFPE) tumor specimen tissues (5 sections per sample). The DNA isolation was performed using a QIAamp DNA FFPE Tissue Kit (Qiagen GmbH; Hilden, Germany) according to the manufacturer’s protocol after deparaffinization using single xylene extraction and rinsing with ethanol 98%. DNA concentrations were evaluated using a Nanodrop ND-1000 microspectrophotometer (Thermo Scientific, Wilmington, DE, USA).

### 4.3. Detection of TERTp Mutations

Detection of *TERT*p mutations was performed by direct sequencing using the Sanger method. Approximately 30 ng of genomic DNA was used as template for polymerase chain reaction (PCR) amplification of 157-bp length fragment of *TERT*p containing the analyzed hotspot sites (−124 and −146 upstream of the ATG start codon). Primers used in the PCR were F-5′-CCCCTTCACCTTCCAGCTC-3′ and R-5′-CAGCGCTGCCTGAAACTC-3′. The reaction was carried out with an initial polymerase activation step at 95 °C for 15 min, followed by 34 consecutive cycles of denaturation at 95 °C for 30 s, primers annealing at 59.7 °C for 30 s, extension at 72 °C for 30 s, and final elongation at 72 °C for 5 min. The PCR product was analyzed for quality by gel electrophoresis and then sequenced on a 3130xl Genetic Analyzer Applied Biosystems (Life Technologies, Carlsbad CA, USA). Briefly, PCR products were enzyme purified with alkaline phosphatase (SAP) and exonuclease I (Life Technologies) and then subjected to standard sequencing using an ABI PRISM^™^ 1.1 BigDye Terminator Cycle Sequencing Ready Reaction Kit (Life Technologies).

### 4.4. Detection of BRAF V600E Mutation

Information regarding the *BRAF* 600 codon status was determined using the following methodology. A fragment of exon 15 of *BRAF* containing codon 600 was amplified by PCR using primers F-5′-TGTTTTCCTTTACTTACTACACCTCA-3′ and R-5′-GCCTCAATTCTTACCATCCA-3′ resulting in amplification of a 160-bp product. The PCR cycling conditions included an initial polymerase activation step at 95 °C for 15 min; 34 cycles of denaturation at 95 °C for 30 s, primer annealing at 56.5 °C for 30 s, extension at 72 °C for 30 s; and the final elongation step at 72 °C for 5 min. After quality confirmation by gel electrophoresis, the PCR products were sequenced as described for detection of the *TERT*p mutations.

### 4.5. Statistical Analysis

The association analysis was performed on categorical data that were summarized as numbers and percentages. Continuous data were summarized as medians and interquartile ranges.

In order to test, whether there were significant differences between analyzed groups of tumors, the Fisher’s exact test and the Mann–Whitney U test were applied. The Fisher’s exact test was used for comparisons in case of categorical variables, whereas the Mann–Whitney U test was used in case of continuous variables. All *p*-values were two sided, and a *p*-value of 0.05 was considered as statistically significant.

Statistical analyses were performed using the R software version 3.4.1 and Gmisc package version 1.4.1 [34,35].

## 5. Conclusions

Although most PTCs represent relatively indolent tumors, some cases develop distant metastases or recurrent disease, or may dedifferentiate into PDTCs or ATCs. There is no definitive answer regarding whether *TERT*p mutations are the key to PTC aggressiveness and dedifferentiation. However, more and more recently published data, including our current study, suggest their association with a poor prognosis. Despite the small number of analyzed PTC samples the frequency of *TERT*p hotspot mutations as well as the association between the *BRAF* V600E mutation and some PTC histopathological factors, observed in this preliminary study, were in concordance with the results of previous reports. Moreover, we identified three novel *TERT*p alterations, which may play a role in PTC aggressiveness. Further functional studies are needed to determine their impact. Although relatively rare, it is clear that *TERT*p mutations in PTCs are an important molecular event and may influence risk stratification and the clinical management.

## Figures and Tables

**Table 1:** Association of *TERT* promoter (*TERT*p) hotspot mutations and the *BRAF* V600E mutation.

***TERT*/*BRAF* Status**	***BRAF* V600E-Positive**	***BRAF* V600E-Negative**	***p*-Value**
	**(*n* = 87)**	**(*n* = 96)**	
***TERT*p hotspot mutation**			0.007
***TERT*p-positive**	13 (14.9%)	3 (3.1%)	
***TERT*p-negative**	74 (85.1%)	93 (96.9%)	
	***TERT*p-Positive**	***TERT*p-Negative**	***p*-Value**
	**(*n* = 16)**	**(*n* = 167)**	
***BRAF* mutation**			0.007
***BRAF* V600E-positive**	13 (81.2%)	74 (44.3%)	
***BRAF* V600E-negative**	3 (18.8%)	93 (55.7%)	

**Table 2:** Association of *TERT* promoter (*TERT*p) hotspot mutations, the *BRAF* V600E mutation, their co-existence, and other *TERT*p alterations with histopathological features of the analyzed papillary thyroid carcinoma (PTC) cohort.

Histopathological Features	No Mutation		*BRAF* + *TERT*p−			*BRAF* − *TERT*p+			*BRAF* + *TERT*p+			*TERT*p_Others		
	No. (%)	No. of Missing Cases	No. (%)	No. of Missing Cases	*p*-Value	No. (%)	No. of Missing Cases	*p*-Value	No. (%)	No. of Missing Cases	*p*-Value	No. (%)	No. of Missing Cases	*p*-Value
**Total No. Of Cases**	**93**		**74**			**3**			**13**			**6**		
**Sex**					0.3			0.23			0.006			1
Female	86 (92.5%)		64 (86.5%)			2 (66.7%)			8 (61.5%)			6 (100.0%)		
Male	7 (7.5%)		10 (13.5%)			1 (33.3%)			5 (38.5%)			0 (0.0%)		
**Age at diagnosis (years)**	50.0 (36.0–58.0)		54.0 (40.2–64.0)		0.024	70.0 (68.0–76.5)		0.005	71.0 (63.0–75.0)		<0.0001	48.5 (34.0–60.8)		0.94
**Tumor diameter (mm)**	7.0 (4.0–14.0)	1	13.0 (8.0–17.8)		<0.0001	12.0 (11.0–13.0)		0.21	25.0 (17.2–31.5)	1	<0.0001	10.0 (3.5–18.8)		0.91
**Histopathological variant**		10		6	0.97			0.041		2	0.14			1
Classical (CPTC)	34 (41.0%)		29 (42.6%)			1 (33.3%)			8 (72.7%)			3 (50.0%)		
Follicular (FVPTC)	23 (27.7%)		21 (30.9%)			0 (0.0%)			1 (9.1%)			2 (33.3%)		
Classical and follicular	16 (19.3%)		12 (17.6%)			0 (0.0%)			0 (0.0%)			1 (16.7%)		
Oxyphilic	5 (6.0%)		3 (4.4%)			2 (66.7%)			1 (9.1%)			0 (0.0%)		
Others	5 (6.0%)		3 (4.4%)			0 (0.0%)			1 (9.1%)			0 (0.0%)		
**pT**		1		1	<0.0001			0.14			<0.0001			0.14
1a	58 (63.0%)		19 (26.0%)			1 (33.3%)			0 (0.0%)			2 (33.3%)		
1b	12 (13.0%)		23 (31.5%)			2 (66.7%)			3 (23.1%)			2 (33.3%)		
2	9 (9.8%)		5 (6.8%)			0 (0.0%)			1 (7.7%)			0 (0.0%)		
3	13 (14.1%)		24 (32.9%)			0 (0.0%)			7 (53.8%)			2 (33.3%)		
4a	0 (0.0%)		2 (2.7%)			0 (0.0%)			2 (15.4%)			0 (0.0%)		
**pT**		1		1	0.002			1			<0.0001			0.23
T1/T2	79 (85.9%)		47 (64.4%)			3 (100.0%)			4 (30.8%)			4 (66.7%)		
T3/T4	13 (14.1%)		26 (35.6%)			0 (0.0%)			9 (69.2%)			2 (33.3%)		
**Lymph node metastases**		14		21	0.42		2	1		6	<0.0001		2	0.16
0	59 (74.7%)		34 (64.2%)			1 (100.0%)			0 (0.0%)			2 (50.0%)		
1a	11 (13.9%)		10 (18.9%)			0 (0.0%)			2 (28.6%)			0 (0.0%)		
1b	9 (11.4%)		9 (17.0%)			0 (0.0%)			5 (71.4%)			2 (50.0%)		
**Infiltration of the tumor capsule**					0.0007			0.51			0.0007			0.6
Yes	19 (20.4%)		34 (45.9%)			1 (33.3%)			9 (69.2%)			2 (33.3%)		
No	74 (79.6%)		40 (54.1%)			2 (66.7%)			4 (30.8%)			4 (66.7%)		
**Angioinvasion**					1			0.26			0.13			1
Yes	8 (8.6%)		6 (8.1%)			1 (33.3%)			3 (23.1%)			0 (0.0%)		
No	85 (91.4%)		68 (91.9%)			2 (66.7%)			10 (76.9%)			6 (100.0%)		
**Multifocality**					0.83			1			0.69			0.59
Yes	14 (15.1%)		12 (16.2%)			0 (0.0%)			1 (7.7%)			0 (0.0%)		
No	79 (84.9%)		62 (83.8%)			3 (100.0%)			12 (92.3%)			6 (100.0%)		

“No mutation” refers to PTCs without the *BRAF* V600E mutation or *TERT*p alterations.

**Table 3:** Characteristics of the study cohort.

*TERT* Status and Histopathological Features	Total		*BRAF* V600E-Negative		*BRAF* V600E-Positive	
	No. (%)	No. of Missing Cases	No. (%)	No. of Missing Cases	No. (%)	No. of Missing Cases
**Total No. of cases**	189		100		89	
**Sex**						
Female	166 (87.8%)		92 (92.0%)		74 (83.1%)	
Male	23 (12.2%)		8 (8.0%)		15 (16.9%)	
**Age at diagnosis [years]**	53.0 (40.0–62.0)		50.5 (37.5–59.0)		57.0 (44.0–66.0)	
***TERT* promoter hotspot mutation (C228T and C250T)**						
Lack of mutation	173 (91.5%)		97 (97.0%)		76 (85.4%)	
Mutation	16 (8.5%)		3 (3.0%)		13 (14.6%)	
**Type of *TERT* promoter alteration**						
Number of negative samples	167		93		74	
C228T	13 (59.1%)		3 (42.9%)		10 (66.7%)	
C250T	3 (13.6%)		0 (0.0%)		3 (20.0%)	
alteration −100 C → T; rs2735943	1 (4.5%)		0 (0.0%)		1 (6.7%)	
alteration −162 C->T	1 (4.5%)		0 (0.0%)		1 (6.7%)	
alteration −77 C->T	2 (9.1%)		2 (28.6%)		0 (0.0%)	
alteration −80 C->T	2 (9.1%)		2 (28.6%)		0 (0.0%)	
**Tumor diameter (mm)**	10.0 (5.0–17.0)	2	8.0 (4.5–14.0)	1	13.0 (8.8–20.0)	1
**Histopathological variant**		18		10		8
classical (CPTC)	75 (43.9%)		37 (41.1%)		38 (46.9%)	
follicular (FVPTC)	47 (27.5%)		24 (26.7%)		23 (28.4%)	
classical and follicular	29 (17.0%)		17 (18.9%)		12 (14.8%)	
oxyphilic	11 (6.4%)		7 (7.8%)		4 (4.9%)	
others	9 (5.3%)		5 (5.6%)		4 (4.9%)	
**pT**		2		1		1
1a	80 (42.8%)		60 (60.6%)		20 (22.7%)	
1b	42 (22.5%)		16 (16.2%)		26 (29.5%)	
2	15 (8.0%)		9 (9.1%)		6 (6.8%)	
3	46 (24.6%)		14 (14.1%)		32 (36.4%)	
4a	4 (2.1%)		0 (0.0%)		4 (4.5%)	
**pT**		2		1		1
T1/T2	137 (73.3%)		85 (85.9%)		52 (59.1%)	
T3/T4	50 (26.7%)		14 (14.1%)		36 (40.9%)	
**Lymph node metastases**						
0	96 (66.7%)		61 (74.4%)		35 (56.5%)	
1a	23 (16.0%)		11 (13.4%)		12 (19.4%)	
1b	25 (17.4%)		10 (12.2%)		15 (24.2%)	
**Distant metastases**		2		2		
0	187 (100.0%)		98 (100.0%)		89 (100.0%)	
**Infiltration of the tumor capsule**						
yes	65 (34.4%)		21 (21.0%)		44 (49.4%)	
no	124 (65.6%)		79 (79.0%)		45 (50.6%)	
**Angioinvasion**						
yes	18 (9.5%)		9 (9.0%)		9 (10.1%)	
no	171 (90.5%)		91 (91.0%)		80 (89.9%)	
**Multifocality**						
yes	27 (14.3%)		14 (14.0%)		13 (14.6%)	
no	162 (85.7%)		86 (86.0%)		76 (85.4%)	

## Abbreviations

ATC	Anaplastic thyroid carcinoma
ETS	E26 transformation-specific
FTC	Follicular thyroid carcinoma
HCC	Hürthle cell carcinoma
LNM	Lymph node metastases
PDTC	Poorly differentiated thyroid carcinoma
PTC	Papillary thyroid carcinoma
PTMC	Papillary thyroid microcarcinoma
TC	Thyroid carcinoma
*TERT*p	*TERT* promoter
